# Civil Practice

**Published:** 1918-10

**Authors:** 


### Civil Practice.

In these times, there is a tendency to emphasize details of military medicine and especially of war surgery. Without minimizing the importance of these matters and without posing as a prophet, let us not forget that no previous war on such a scale as to draw heavily on the population, has ever lasted for a considerable period as compared with the periods of peace. We need not raise the question as to whether this will or will not be the final great war. Let us not forget the numerically greater incidence at present of non-military affections nor overlook the harm of neglecting interest in the routine problems of practice among a peaceful population.

